# Clinical Lectures on Typhoid Fever, and Diseases of the Heart

**Published:** 1860-03

**Authors:** N. S. Davis


### CLINICAL REPORTS.

Mercy Hospital. Female Ward. Service of Dr. N. S. Davis.

Case 1st.—*Typhoid Fever.* Those members of the class in attendance on the clinical instruction given in this Institution during the month of October, had an opportunity of seeing many cases of continued fever in all stages of their progress.

The case now before you is that of a woman, aged 25 years, native of Ireland, who was admitted into the hospital yesterday. She had been previously confined to her bed ten or twelve days. You observe that the expression of her countenance is dull; the face suffused with a dark flush; the lips dry and of a purplish color; the tongue covered in the middle with a dry, brown coat; the skin dry and slightly elevated in temperature; the abdomen full and tympanitic; the bowels are evacuated from three to six times in the twenty-four hours, the discharges being thin and brown, with some whiteish flakes; the pulse 110 per minute and weak; hearing obtuse, and mind dull with disposition to delirium during the night. From this group of symptoms it is easy to determine that the patient is laboring under an attack of continued fever, of the typhoid or enteric variety. The continuous low grade of fever, the state of the tongue, the tympanitic abdomen, with characteristic intestinal discharges, are sufficient to determine the nature of the disease. It is already at the commencement of the third week in its progress, and you find the symptoms indicating two primary and important pathological lesions, strongly developed. The first of these lesions consists in a depressed or impaired state of the properties of both solids and fluids, throughout the whole system. Thus, as I have previously shown you, the blood is darker color, and the fibrin less coagulable than in health; and the susceptibility of all the organized structures is impaired, as strongly indicated in this patient by the dark, dingy hue of the face; the dulness of intellect and special senses; the awkwardness and irregularity of muscular movements; the want of vigor in the movements of circulation and respiration; and the general diminution of secretion. The second lesion is the well known disease of the agminated glands of the ilium, here plainly indicated by the dry tongue, the tympanitic abdomen, and the brown liquid evacuations.

We find both these pathological lesions in all cases of the enteric variety of typhoid fever; but they present very variable degrees of development in different cases. In some cases the *first* so strongly predominates that the life of the patient is endangered from the extreme impairment of the properties of the solids and fluids, or what is more commonly called "early and severe prostration;" with only slight symptoms of intestinal disease. While in other cases the first is only slight, and the second or intestinal lesion predominates from the beginning of the fever, and the life of the patient is put in jeopardy only by the extent of the consequent ulcerations in the glands of the intestine.

In the patient before you, gentlemen, both these pathological conditions are sufficiently developed to render the prognosis doubtful, though we think she will recover.

*Treatment.*—On this subject we cannot agree with those who tell us that typhoid fever is a self-limited disease, destined to run a definite course, and therefore the whole duty of the physician consists in adopting judicious hygienic regulations, and paliating symptoms. On the contrary, we think the pathological conditions here present afford as well defined indications for the use of remedial agents as do the paroxysms of an intermittent. If you look upon this patient with the eye of an intelligent and rational pathologist, you will see in the dark hue and impaired quality of the blood an indication for the use of some agent that will increase the capacity of that fluid for absorbing oxygen and parting with carbonic acid gas, thereby restoring its normal color and arresting the further deterioration of its qualities.

You will see with equal readiness, in the general impairment of those properties of the solids which we call susceptibility and vital affinity, the symptoms of which were pointed out in detail a few minutes since, an indication for the use of some remedial agent calculated to increase and sustain these properties. Finally, you see in those symptoms that point directly to a well-known lesion in the mucous membrane of the ilium, an indication for the use of such agents as will allay the sensibility, and increase the tone of the capillaries of the intestine, and thereby arrest the processes of softening and ulceration of the glands.

Here, then, you have there well-defined indications for treatment, founded on the actual pathological conditions of the patient. But what are the means for fulfiling these several indications? If we remember rightly, it was M. Claude Bernard, who a few years since performed a series of experiments to determine the influence of different agents on the capacity of the blood for absorbing oxygen, which resulted in showing that chloride of sodium, chlorate of potassa, and several other salts, uniformly increased such capacity; some of them in a striking degree. It had long been known that some of these salts possessed the power to change a clot of venous blood to an arterial color, and to retard the dissolution of its constituents. Taking the hint from these facts, we commenced four or five years since to use the chloride of sodium and chlorate of potassa to fulfil the first indication, just pointed out, in the treatment of all the low forms of continued fever. And the more we have used them the more confidence have we felt in their remedial value.

For fulfiling the second indication, the profession have long looked to the class of diffusable stimuli, at the head of which stand the various alcoholic beverages. But the results of their use have not been altogether satisfactory. The reason for this can be readily discovered by studying closely their *modus operandi* upon the human system. By such study it will be found that instead of exalting the elementary properties of the organized structures of the body generally, they simply exhilerate or excite the nervous tissues, while they actually lessen the interchange of carbonic acid gas for oxygen by respiration. Hence they are calculated to fulfil the indication in question very imperfectly at best. If you turn your attention a moment to the great field of nature, you will see that oxygen is the great excitor or supporter of the properties and molecular actions of all living organized matter. And its power to excite all the organic movements of the human system will be known. It is evident, therefore, that if in a case like this before us we could increase the quantity of oxygen in the blood, it would constitute the most efficient remedy known for increasing the depressed susceptibility and renewing those organic movements which are now so sluggish. As we have already explained, certain saline remedies do possess the power to increase the capacity of the blood for absorbing oxygen. Hence when introduced into the system as medicines, they not only improve the color and preserve the integrity of the blood itself, but by increasing the quantity of oxygen absorbed in the lungs, they supply the most universal and efficient excitor of the vital properties and movements with which we are acquainted. Thus the same remedial agents that directly fulfil the first indication, indirectly fulfil the second also. These same considerations shew you why it is so important to keep the room of the continued fever patient well ventilated or supplied with pure air. The third indication plainly requires the use of such agents as will combine the properties of an anodyne with a peculiar local stimulant or astringent, such as opium with nitrate of silver, or sulphate of copper, or oil of turpentine; more especially in the middle and advanced stages of the disease. In accordance with these views we shall prescribe for this patient 8 grs. of chlorate of potassa dissolved in water, every four hours; and a teaspoonful of the following emulsion two hours after each of the doses of the chlorate:

℞ Ol Terebinth. ʒ ii.

Tinct. Opii, ʒ ii.

Pulv. G. Arabac. } aa. ʒ iii.

Sacchar. Alba. }

Rub together thoroughly and add

Mint Water, ℥ ii.

She must also be fed regularly with beef-tea well seasoned with salt, or milk-porridge, made by adding wheat flour to boiling milk.*

Case 2d.—This woman is also laboring under typhoid fever, complicated with a low grade of pneumonic inflammation in the right lung. The case was fully presented to you a few days since, when you individually examined the affected lung with the stethescope. As you now note her progress, we simply remark that all her symptoms are improving, and that the treatment previously prescribed will be continued, with the addition of a blister to the right side of the chest.

Case 3d.—*Syphilitic Ecthyma, with ulceration of the fauces*

We will detain you a short time longer this morning to examine this woman, native of Ireland, aged about 35 years; naturally of good constitution, but now slightly anemic, with an ugly looking eruption upon her face. In looking closely at this eruption you will see that each sore has an elevated base, surrounded by a dark red areola, the cuticle elevated in the centre, and filled with pus. They vary in size from the diameter of a pea to that of a dime. Nearly all of them have a thick brownish yellow scab in the centre. They evidently belong to the class of pustular diseases, and of the variety called Ecthyma. They are distinguished from Impetigo by their larger size, more conical form, and the thicker scabs. They differ still more from Rupia by the yellowish color of the scabs, and the appearance of pus surrounding the scab, between its edge

* The above treatment was strictly carried out, and the patient convalesced in one week after admission, or three weeks after taking to her bed.

and the sound skin, while in the latter disease the scabs are quite black and closely adherent at their edges to the surrounding skin.

If you look into the fauces of the patient you will see a large ulcer which has penetrated entirely through the soft palate immediately above the uvula. The dark red color around this ulcer, like that around the eruption on the face, indicates the existence of a syphilitic influence. Hence we shall put the patient upon the use of the following prescription:

℞ Tinct. Cinchonæ ℥ ii.

Hydrarg. Chlor. Corrosiv. 1 gr.

Mix—take one teaspoonful before each meal-time and at bedtime. We will also touch the ulcer in the throat with a stick of nitrate of silver every other day. After the patient has taken the mercurial until its effects upon the gums can be seen in a very slight degree, we shall substitute therefor the iodide of potassa in pretty large doses, and continue it until the disease appears to be entirely removed, which usually does not occur sooner than six or eight weeks.

---

### CLINIQUE OF PROF. DAVIS IN THE MEDICAL DEPARTMENT OF LIND UNIVERSITY.

Saturday Afternon, Jan. 28th, 1860.

Case 1st. *Disease of the Heart.*—Gentlemen, the patient who presents himself before you is a native of Ireland, aged about 35 years, a blacksmith by trade, and possessed of a well developed physical frame. You observe that his breathing is short and hurried, much like one who has been running; his face and lips rather pale and slightly bloated; his tongue covered with a whiteish fur; his skin above the natural temperature and dry; his pulse moderately full, hard, and 100 per minute; and he complains of a great sense of fulness or oppression in the chest, with a pretty severe dull pain in the cardiac region, increased to a sharp or acute quality on taking a full breath, or coughing. He has severe paroxysms of coughing, and is unable to lie down in a horizontal position without producing a great sense of suffocation, and is consequently obliged to spend his nights with the body in an erect or semi-erect position.

The pain in the cardiac region, accompanied by some fever, commenced about two weeks since, and very soon after an injudicious exposure to wet and cold. These general symptoms, and especially the pain in the region of the heart, coupled with the disturbance of circulation and respiration, would lead us to suspect the existence of cardiac inflammation. To determine this with certainty we must make a physical exploration of the chest by means of auscultation and percussion. On removing the covering from the left side of the chest, and placing the hand over the cardiac region, the impulse of the heart was found much stronger than natural, and percussion shewed that it occupied a larger space. Application of the stethescope over the cartilages of the ribs covering the right side of the heart revealed a well marked friction or rubing sound with each systole of the venticles, and also a rough bellows murmur. On moving the stethescope a little upwards and to the left, so as to rest over the base of the heart, the bellows murmur became more plain, very rough and prolonged, so as to completely suppress the short second sound. The signs thus elicited, clearly indicate not only the existence of cardiac disease, but its existence in a severe and complicated form. The friction sound indicates a recent and still existing inflammation of the pericardium, with more or less plastic effusion upon the surface of the membrane; while the rough and prolonged qualities of the bellows murmur, the dulness over a larger space than natural, with a strong and sustained impulse, certainly indicate considerable hypertrophy of the ventricles with disease of the semi-lunar valves. From the loud and harsh qualities of the murmur and the hypertrophy, it is evident that the disease of the valves has been existing a considerable time previous to the date of the patient's present illness.

He now explains this by informing me that he had a severe attack of rheumatism, accompanied by pain in his left breast two years since; and that he has been seriously troubled with shortness of breath and heavy irregular beating of the heart, especially on taking exercise, from that time to the present.

From all these facts we infer that the patient was attacked with rheumatic endocarditis two years since, which resulted in thickening of the semi-lunar valves, and the consequent gradual development of hypertrophy of the muscular structure of the ventricles. While the injudicious exposure to wet and cold two weeks since caused the supervention of a sub-acute pericarditis which still exists. Placing the patient in an easy position, each member of the class was permitted to take the stethescope and listen to all the morbid sounds that the case presented.

*Prognosis.*—The prognosis in recent attacks of inflammation of the heart, whether endocardial or pericardial, may be regarded as favorable. And so far as the pericardial disease is concerned in this case, we think it can be removed in a few days by appropriate treatment; though, as sometimes happens, the effusion of plastic lymph may cause adhesion of greater or less extent between the surfaces of the inflamed membrane. I have observed two cases, in which such adhesions existed so exten sively as to unite the pericardium closely and firmly to the whole exterior surface of the heart, thereby completely obliterating the pericardial sac.

One of these was a recent case, and in addition to the adhesions, there was present intense redness and all the marks of acute inflammation in all the structures of the heart. The other case had been one of long standing, and the adhesions were so firm that it was with difficulty that the pericardium could be torn from the surface of the heart. If we are right in the supposition that this patient was attacked with endocarditis two years since, and that the thickened condition of the valves which now gives rise to the harsh bellows murmur to which you have listened, is the result of that inflammation, it is quite certain that such thickening and the hypertrophy consequent upon it, have become too permanent to admit of removal by any remedial agents known to the profession. On the contrary, the continuance of the valvular obstruction, will cause a gradual increase of the hypertrophy, until at length the patient becomes wholly unable to exercise; dropsical effusions supervene, and life is cut short.

*Treatment.*—The first object to be accomplished in the treatment of this patient, is to remove the recent inflammation of the pericardial membrane. This must be done by a prompt and judicious use of sedatives, alteratives, and counter-irritants. Had the patient come under our care during the first two or three days after the commencement of the present attack, we might have deemed it necessary to have taken at least one free bleeding from the arm. But two weeks having now elapsed and the patient feeling already debilitated, we do not deem venesection necessary. Consequently we shall endeavor to control the circulation by the following, viz:

| | | | |
|---|---|---|---|
| ℞ | Spts. Nit. Dulc. | a.a. | ℥ i. |
| | Tinct. Opii et Camph. | | |
| | Tinct. Verat. Viride | | ʒ i. |

Mix, and give a teaspoonful every four hours, diluted with water.

To change rapidly the tendency to plastic effusion, and consequent adhesions, and destroy the inflammatory process, we shall also direct one of the following powders to be taken between each of the doses of the sedative mixture, viz;

| | | |
|---|---|---|
| ℞ | Hydrarg. Chlor. Mit. | 15 grs. |
| | Pulv. Opii. | 12 grs. |
| | Nitras Potassa, | ℈ ii. |

Mix and divide into six powders.

By giving the opium in full doses, we shall not merely relieve the pain and restlessness of the patient, but we shall overcome that important element in all inflammations, which we denominate irritability, or more properly, an exaltation of the susceptibility of the inflamed structure; and thereby aid much in destroying the inflammatory process.

We shall continue these remedies until the calomel produces a slight effect on the gums, and then interpose a cathartic.

After the bowels have been freely moved, the previous remedies may be resumed with the calomel omitted; and a blister may be placed over the cardiac region.

It is probable that under the influence of these remedies, the present pericardial inflammation will be removed in from four to six days; but the valvular obstruction and hypertrophy, with some general debility will remain. If so, all active treatment by medication may be discontinued. The patient must be instructed to wear flannel next the skin; avoid sudden atmospheric changes; make his exercise, mental and physical, as quiet as possible; avoid the use of stimulants, and highly seasoned articles of food.

In addition to these hygienic regulations, the increase of hypertrophy may be retarded by a judicious use of the milder sedatives, such as Digitalis, Gelsemin, and Acetate of Lead. But as this patient will probably return to the clinic next week, we will not comment on his case further at present.

Two children were presented to the class in this clinique, one afflicted with Bronchitis, and the other with Chronic Ague, but as the space allotted to these reports is already full, we must defer giving the substance of the remarks made concerning them until a future time.

